# Effect of neem leaf extract (*Azadirachta indica*) in reducing the degree of parasitemia and apoptosis in C57BL mice with cerebral malaria

**DOI:** 10.14202/vetworld.2024.1497-1503

**Published:** 2024-07-10

**Authors:** Zainabur Rahmah, Kautsar Citra Nirmala, Ach Nashichuddin, Riskiyana Riskiyana, Alvi Milliana, Nurfianti Indriana, Lina Fitria Astari, Prida Ayudianti, Munawar Kholil

**Affiliations:** 1 Department of Parasitology, Faculty of Medicine and Health Sciences, Universitas Islam Negeri Maulana Malik Ibrahim Malang, Indonesia; 2 Medicine Study Program, Faculty of Medicine and Health Sciences, Universitas Islam Negeri Maulana Malik Ibrahim Malang, Indonesia; 3 Department of Mathematics, Faculty of Science and Technology, Universitas Islam Negeri Maulana Malik Ibrahim Malang, Indonesia; 4 Department of Neurology, Faculty of Medicine and Health Sciences, Universitas Islam Negeri Maulana Malik Ibrahim Malang, Indonesia; 5 Department of Microbiology, Faculty of Medicine, and Health Sciences, Universitas Islam Negeri Maulana Malik Ibrahim Malang, Indonesia; 6 Department of Obstetrics and Gynecology, Faculty of Medicine, and Health Sciences, Universitas Islam Negeri Maulana Malik Ibrahim Malang, Indonesia; 7 Department of Child Health, Faculty of Medicine, and Health Sciences, Universitas Islam Negeri Maulana Malik Ibrahim Malang, Indonesia; 8 Department of Dermatology and Venereology, Faculty of Medicine, and Health Sciences, Universitas Islam Negeri Maulana Malik Ibrahim Malang, Indonesia; 9 Department of Agriculture Product Technology, Politeknik Negeri Ketapang, Ketapang, Indonesia

**Keywords:** apoptosis, *Azadirachta indica*, cerebral malaria, neem leaves, parasitemia

## Abstract

**Background and Aim:**

Brain malaria, which results from *Plasmodium falciparum* infection, is responsible for substantial fatalities and health issues. These processes, including cytoadherence, rosetting, and sequestration, induce an immune response, hypoxia, brain microvascular obstruction, disruption of the blood-brain barrier, and cell death. Parasitemia level can reveal the presence of infection and its association with apoptosis-related genes. Neem (*Azadirachta indica*) leaves with antimalarial properties could replace ineffective Indonesian malaria medications. This study was designed to evaluate the impact of neem leaf extract on cerebral malaria-induced parasitemia and neuron cell apoptosis in mice through an *in vivo* approach.

**Materials and Methods:**

13–16 weeks old C57BL mice received infection by *Plasmodium berghei* strain ANKA. Parasitemia was estimated daily from the mice’s tail blood. 8 mg, 12 mg, and 16 mg of a 96% ethanolic neem leaf extract were orally given for 6 days. Healthy, positive, and negative controls were included for treatment comparisons. On the 7^th^ day, brain tissue was analyzed for (p > 0.05) gene expression. Through immunohistochemistry, both cell apoptosis in neurons expressing caspase-3 within a brain sample and the degree of parasitemia in a blood smear were assessed. The Pearson correlation test and one-way analysis of variance were employed to analyze the data.

**Results:**

Neem leaf extract reduces parasitemia and neuron cell apoptosis at multiple dosages (p < 0.000). Apoptosis in brain neurons and parasitemia show a strong positive correlation (r = +0.939). Neem leaf extract at doses of 12 and 16 mg was the most effective in reducing parasitemia levels and causing cell death.

**Conclusions:**

Neem leaf therapy significantly reduced the degree of parasitemia and cell apoptosis in C57BL mice compared with the control group without treatment (p = 0.05). This shows that neem leaves have the potential to be a candidate drug for malaria.

## Introduction

Eighty-seven countries worldwide suffer from malaria, which poses a threat to public health [1]. The World Health Organization (WHO) reported 241 million malaria cases worldwide in 2020 [2]. The elimination of malaria is the government of Indonesia’s 2030 goal. Because of the region’s climate and geographic position, which play a major role in the persistence of malaria and provide considerable obstacles to efforts to control and eliminate the disease, Muara Enim Regency in South Sulawesi continues to see extremely high rates of malaria transmission. [3]. Malaria is transmitted through the *Plasmodium* parasite through the bites of female *Anopheles* mosquitoes [4]. *Plasmodium falciparum* is the most deadly species, accounting for 60%–70% of fatalities [5]. Determining the parasitemia level aids in diagnosing *P. falciparum* parasite infection. The degree of parasitemia can be determined by counting the number of infected erythrocytes in a wide range of view during a blood smear examination [6]. Cerebral malaria, as a result of *P. falciparum* infection, is one of the most frequent malaria emergencies [7]. Neurological symptoms include seizures, coma, and altered consciousness, among others [8]. The mortality rate from cerebral malaria in Indonesia ranges between 21.5% and 30.5% [9].

*P. falciparum* erythrocyte membrane protein 1 on erythrocytes binds to endothelial cell surface proteins, such as intercellular adhesion molecule 1 (ICAM-1), vascular cell adhesion molecule 1, and a cluster of differentiation 36 (CD36), causing cytoadherence and sequestration, which is the mechanism underlying cerebral malaria [10]. CD8+ T lymphocytes induce cell death in response to microvasculature blockage. Other cell types are less likely to undergo apoptosis than neurons [11, 12]. In addition, heme oxygenase and hemozoin (Hz), which are byproducts of parasite metabolism, contribute to apoptosis [13].

Indonesia is a nation with a high malaria rate that seeks suitable and efficient treatment. However, there was a decline in effectiveness and possibly treatment resistance [14]. Neem (*Azadirachta indica*) is a plant species that is widely distributed and can be used as an alternative treatment. This plant’s Biologically active substances include azadirachtin, the main active substance in the seeds and leaves, flavonoids, alkaloids, triterpenoids, phenolics, carotenoids, steroids, and ketones [15].

This study was designed to evaluate the impact of neem leaf extract on cerebral malaria-induced parasitemia and neuron cell apoptosis in mice through an *in vivo* approach.

## Materials and Methods

### Ethical approval

The Health Research Ethics Committee FKIK UIN Maulana Malik Ibrahim Malang has granted ethical permission for this study under the following number: No. 20/20/EC/KEPK-FKIK/08/2023. This study complies with ARRIVE (https://www.nc3rs.org.uk/arrive-guidelines) and the guide for the care and use of laboratory animals (https://www.ncbi.nlm.nih.gov/books/NBK54050/).

### Study period and location

This study was conducted from February 2023 to December 2023 in the Parasitology Laboratory, Faculty of Medicine and Health, Universitas Islam Negeri Maulana Malik Ibrahim Malang, and the Parasitology Laboratory, Anatomical Pathology Laboratory, Faculty of Medicine, Universitas Brawijaya, East Java, Indonesia.

### Animals

Twenty-four male C57BL mice weighing between 20 g and 30 g were used; they were 13–16 weeks old. The mice were kept in the study laboratory in housing that ranged from 20°C to 24°C, with a 12-h light/dark cycle. They also had unrestricted access to food and laboratory water.

### Materials

The *Plasmodium berghei* ANKA strain inoculation was obtained from the Parasitology Laboratory of the Faculty of Medicine, Universitas Brawijaya. The Caspase 3 kit was obtained from Santa Cruz Biotechnology, Dallas, Texas**.**

### Preparation of the experimental animals

This purely experimental research used a post-test-only control group method *in vivo* using brain samples from C57BL mice. These mice can present with clear symptoms and pathological features. Four mice were allocated to each of the six treatments. The groups consisted of healthy controls (not infected and not treated), negative controls (infected and not treated), and positive controls (treated with dihydroartemisinin piperaquine [DHP] 0.02496 mg), treatment 1 (neem leaf extract 8 mg), treatment 2 (neem leaf extract 12 mg), and treatment 3 (neem leaf extract 16 mg).

### Making neem leaf extract

The region of Madura, East Java’s Pamekasan, was the source of neem leaves. The Batu Malang Herbal Materia Medica Laboratory UPT Number: 000.9.3/2779/102.20/2023 identified neem plants. As part of the extraction procedure, 100 g of neem leaves are first dried and then macerated for 48 h with air and 96% ethanol. To obtain a concentrated extract, the filtrate was heated to 40°C after filtration and then evaporated. Neem leaf extract was diluted with 0.5% carboxy methyl cellulose solution.

### Neem leaf simplicia

Fresh and healthy neem leaves were chosen and thoroughly washed under running water and then set aside to dry. The next step was to dry it in an oven set at 50°C for 3 days to give it a basic shape. Next, the simplicial leaf neem was ground into a powder. To create fine simplicia powder, the simplicia powder was sieved.

### Phytochemical test of the neem leaf extract

Two grams of neem leaf extract was dissolved in 20 mL of distilled water. Then, it was heated for approximately 15 min over a Bunsen and poured into two test tubes. The sample was split into two portions, one for testing with flavonoids (quercetin), and the other with triterpenoids (azadiractin). Three drops of concentrated HCl and a pinch of magnesium powder were added to the flavonoid sample for the test. The solution turned orange/brick red/orange/dark red on the addition of the flavonoid (+). Three drops of Bouchardat reagent turned the samples with triterpenoid (+) to brown precipitate.

### Inoculation of the *P. berghei* strain ANKA

Using Giemsa-stained blood smears, parasitemia was computed after donor mice were injected intraperitoneally with 1 × 10^6^/mL of the *P. berghei* strain ANKA containing liquid nitrogen. The parasitemia level should be between 5% and 8% before the mice are inoculated. The Inoculation took place at the Universitas Brawijaya Faculty of Medicine’s Parasitology Laboratory.

### Examination of the degree of parasitemia

The parasitemia level was determined through 6-day long examination of Giemsa-stained tail blood samples. One thousand healthy erythrocytes were examined under a 1000× magnification microscope (Olympus, Japan) for the presence of parasites, the number of which was then calculated and multiplied by 100%.

### Examination of apoptosis (expression of caspase 3) in the brain

2–3 mm thick slices of paraffin-blocked brain tissue were rehydrated by alcohol following xylol deparaffinization. Endogenous peroxidase stopped shear by reacting with methanol, phosphate-buffered saline (PBS), and 30% H_2_O_2_ to terminate antibody binding. 0.1% Triton X-100 and bovine serum albumin buffer were applied during incubation. Afterward, the slide was treated with an anti-caspase antibody (SC-56053, Santa Cruz Biotechnology) recognizing caspase-3 (31A1067). The slides were washed with PBS. Subsequently, the slides were incubated with 3,3′-diaminobenzidine (DAB) reagent, followed with hematoxylin staining and ended with mounting medium. 1000× magnification and 10 fields of view were used to examine the slides under the microscope. When caspase-3 is expressed, the nucleus and cytoplasm appear blackish-brown. Slides were produced in the Anatomic Pathology Laboratory.

### Statistical analysis

Data were analyzed using SPSS version 26 (IBM Corp., NY, USA). Normality and homogeneity were evaluated using the Shapiro Wilk method and the Levene test method, respectively (p > 0.05). One-way analysis of variance (ANOVA) with post-hoc least significant difference (LSD) (p < 0.05) was used for hypothesis testing. Pearson correlation test was used to examine the relationship between parasitemia and brain neuron cell apoptosis.

## Results

### Phytochemical testing of neem leaves (*A. indica*)

Phytochemical tests on neem leaves were conducted at Materia Medica Batu Malang in East Java. The phytochemical composition of *A. indica* leaf extracts is presented in Table-1.

**Table-1:** Phytochemical screening of leaf extracts of *A. indica*.

Phytochemicals	96% Ethanol	Aqueous
Phenols	+	+
Flavonoids	+	+
Tannins	+	+
Saponins	+	+
Alkaloids	+	+
Terpenoids	+	+
Steroids	-	-

+=Present, -=Absent, *A. indica=Azadirachta indica*

### Effect of neem leaf extract therapy (*A. indica*) on the degree of parasitemia induced by cerebral malaria

**Figure-1:**
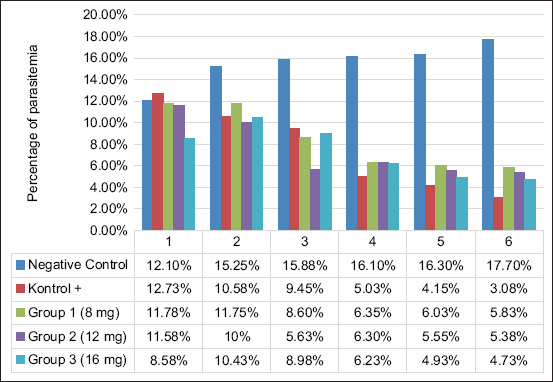
Graph of average degree of parasitemia during 6 days of treatment.

Parasitemia levels were monitored daily for 6 days to assess the treatment’s effect. The negative control group was the only one that did not show a drop in every therapy. The parasitemia levels were 3.08% for DHP 0.02496 mg, 5.83% for treatment 1 (8 mg), 5.38% for treatment 2 (12 mg), and 4.73% for treatment 3 (16 mg). In contrast, the negative control group continued to exhibit growth, as depicted in Figure-1.

**Figure-2:**
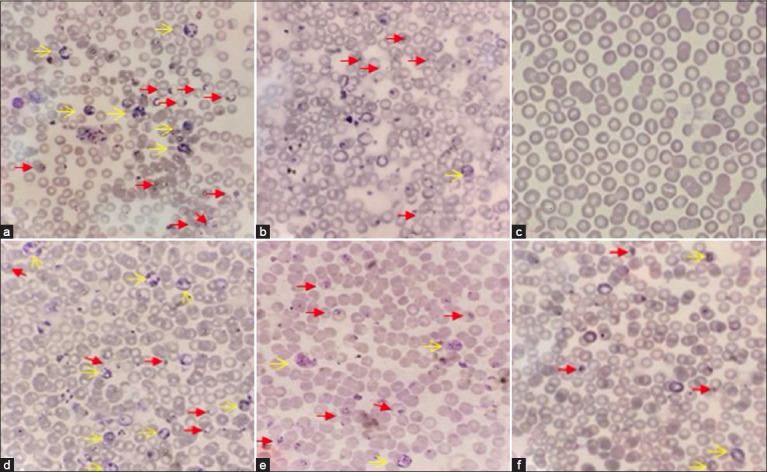
Observation of parasitemia in mice induced by *Plasmodium berghei*. (a) Negative control, (b) Positive control (DHP), (c) Healthy controls, (d) Treatment group 1(neem leaves extract dose of 8 mg), (e) Treatment group 2 (neem leaves extract dose of 12 mg), and (f) Treatment group 3 (neem leaves extract dose of 16 mg). The trophozoite phase is indicated by the thick red arrow, and the schizont phase is indicated by the thin yellow arrow (Examined by Olympus microscope under 1,000× magnification).

The presence of the trophozoite and schizont stages signifies the intensity of malarial infection. In mice, Figure-2 shows the presence of *P. berghei*-induced parasitemia.

Both tests showed no significant difference in parasitemia levels (p > 0.05), but the one-way ANOVA test confirmed this with p < 0.000 significance. The neem leaf extract group showed a markedly distinct decrease in parasitemia compared to the control group. The LSD test did not show any significant differences among treatments 1, 2, and 3 (p values were 0.050, 0.823, and 0.987, respectively). The third group exhibited skills similar to the positive control, while treatment group 2 demonstrated greater effectiveness. In Table-2, the results of the LSD *post hoc* test on parasitemia degree are displayed.

**Table-2:** LSD *post hoc* test (parasitemia variable).

Treatment group	Mean ± standard deviation	Significance value				

		Positive control	Negative control	Group 1 (8 mg)	Group 2 (12 mg)	Group 3 (16 mg)
Positive control	7.33 ± 0.45	-	0.000*	0.050	0.823	0.987
Negative control	15.55 ± 0.66	0.000*	-	0.000*	0.000*	0.000*
Group 1 (8 mg)	8.32 ± 1.02	0.050	0.000*	-	0.077	0.052
Group 2 (12 mg)	7.44 ± 0.42	0.823	0.000*	0.077	-	0.836
Group 3 (16 mg)	7.34 ± 0.53	0.987	0.000*	0.052	0.836	-

(*) shows the significance value. LSD=Least significant difference

### Beneficial impact of *A. indica* neem leaf extract on brain neuron cell apoptosis caused by cerebral malaria

**Figure-3:**
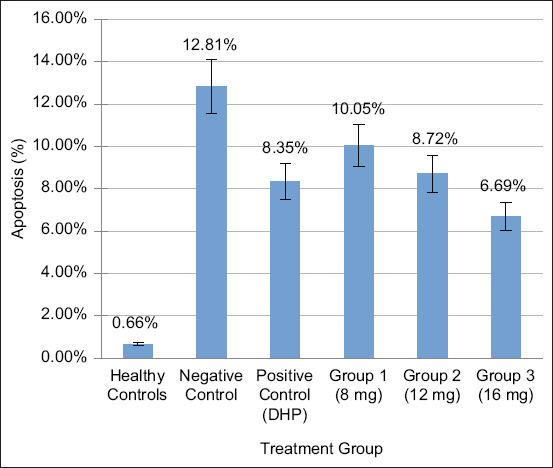
Average neuron cell apoptosis on day 6 of the cerebral malaria group.

Ten fields of view were used to observe caspase-3 expression in apoptotic brain neuron cells. Each slide’s apoptosis percentage was calculated by totaling the number of apoptotic cells and dividing it by the sum of apoptotic and non-apoptotic cells then multiplying the result by 100%. The average of every therapy was calculated. On day 6, the average neuron cell apoptosis in the cerebral malaria group is shown in Figure-3.

**Figure-4:**
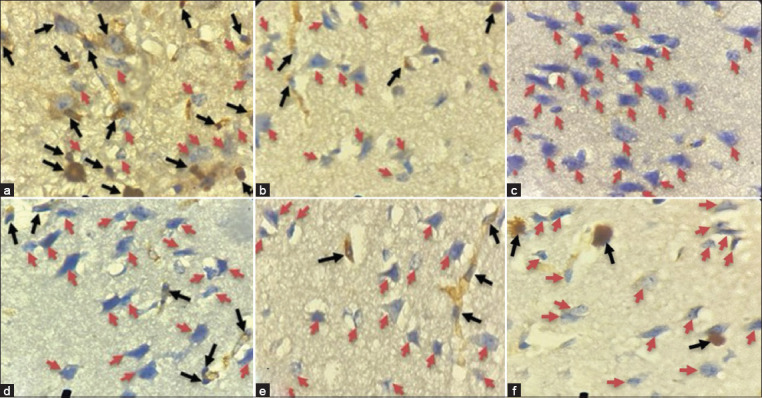
Observation of apoptosis of brain neuron cells marked by the expression of caspase 3. (a) Negative control, (b) Positive control (DHP), (c) Healthy controls, (d) Treatment group 1(neem leaves extract dose of 8 mg), (e) Treatment group 2 (neem leaves extract dose of 12 mg, and (f) Treatment group 3 (neem leaves extract dose of 16 mg). Black arrows indicate neuron cells undergoing apoptosis, while healthy neuron cells are red (Examined by Olympus microscope under 1,000× magnification).

Among neuronal cells undergoing apoptosis, the negative control showed the greatest caspase-3 expression (12.81%). The percentage of neuronal cells undergoing apoptosis was lowest in treatment 3 (6.69%) compared to treatments 1 (10.05%) and 2 (8.72%) after being exposed to neem leaf extract. About 8.35% of positive controls and 0.66% of healthy controls had the least gene expression. The nucleus and cytoplasm, with a blackish-brown hue, display Caspase-3 activity. The expression of caspase 3 in Figure-4 signifies neuron cell apoptosis in the brain.

The neuronal cell apoptosis normality and homogeneity test results were significant (p < 0.05). A one-way ANOVA test with a significance value (p < 0.000), meaning that there was a significant difference in the reduction of cell apoptosis in the group given neem leaf extract. In the neem leaf extract group, cell apoptosis was reduced more than in the control group. The LSD *post hoc* test revealed p-values of 0.000, 0.038, and 0.000 for treatments 1, 2, and 3, respectively, which indicated significant differences between the treatment and control groups. The degree to which these three treatments lessen neuron apoptosis is comparable. Among the groups, including the positive control group (DHP), treatment group 3 demonstrated the greatest capacity. The LSD *post hoc* test was performed on the brain neuron cell apoptosis data of C57BL mice with cerebral malaria.

### Correlation between reducing the degree of parasitemia and apoptosis of brain neuron cells in C57BL mice in cerebral malaria induction

The correlation between parasitemia level and cell apoptosis was strong and significant (r = +0.939, p = 0.000). High parasitemia levels directly cause an increase in brain neuron cell apoptosis. In Table-3, the Pearson correlation between neuron cell apoptosis and parasitemia is analyzed.

**Table-3:** Pearson correlation analysis of neuron cell apoptosis and parasitemia.

Correlation coefficient (rs)	p-value	N
+0.939	0.000	20

## Discussion

The trophozoite stage of the malaria life cycle is represented by the image of infected erythrocytes (Figure-2), and as this stage of infection is marked by a heavy infection, the likelihood of parasitemia is also high [16]. Treatment 2 (12 mg) exhibited the greatest parasitemia reduction (6.20%). Treatment 1 (8 mg) and treatment 3 (16 mg) displayed a reduction of 5.95% and 3.85%, respectively. Certain hereditary variables, dosage amounts, and delivery techniques that affect toxicity susceptibility and treatment response may cause this [17]. Previous research by Afolabi *et al*. [18] has shown that the administration of 600 mg/kg neem leaf extract to *P. berghei*-infected albino mice gives the most potent reduction in parasitemia.

Neem leaves’ flavonoids, notably those with antiplasmodial properties and putative capability to suppress fatty acid synthesis, contribute to diminished parasitemia in malaria [19]. Azadirachtin, a chemical, halts *P. falciparum* gamete development during exflagellation. Phenols and saponins inhibit heme polymerization to deliver antimalarial effects which are harmful to humans [20–22]. The parasitemia level in the 12 mg medication group grew from 8.58% to 10.43% on the 2^nd^ day (Figure-1) *P. berghei*’s asynchronous intraerythrocytic development and variable durations explain why this occurs. This escalates the schizogony cycle and amplifies the parasitemia acutely [23, 24].

In the DHP-treated control group, parasitemia and cell apoptosis levels decreased. The WHO advises using DHP, a treatment composed of artemisinin and piperaquine, to hinder apoptosis by obstructing Hz formation through heme detoxification and decrease parasite-induced parasitemia through enzyme inhibition during DNA and RNA synthesis [25, 26]. The reduction in cell apoptosis is depicted in Figure-3 as neem leaf extract dosage increases. With a 6.69% decrease in apoptotic cells, treatment 3 (16 mg) had the greatest impact, as shown in Table-4. The neem leaf compounds flavonoids, tannins, and saponins are believed to suppress the expression of proinflammatory cytokines, which then inhibit nuclear factor-kappa B (NF-kB) activation and subsequent cytochrome c release [27].

**Table-4:** LSD *post hoc* test results (neuron cell apoptosis variable).

Treatment group	Mean ± standard deviation	Significance value				

		Positive control	Negative control	Group 1 (8 mg)	Group 2 (12 mg)	Group 3 (16 mg)
Positive control	8.35 ± 0.13	-	0.000*	0.000*	0.038*	0.000*
Negative control	12.81 ± 0.19	0.000*	-	0.000*	0.000*	0.000*
Group 1 (8 mg)	10.05 ± 0.22	0.000*	0.000*	-	0.000*	0.000*
Group 2 (12 mg)	8.72 ± 0.20	0.038*	0.000*	0.000*	-	0.000*
Group 3 (16 mg)	6.69 ± 0.35	0.000*	0.000*	0.000*	0.000*	-

(*) shows the significance value. LSD=Least significant difference

Azadirachtin, a triterpenoid compound, shields neurons directly exposed to proinflammatory cytokines, providing an anti-inflammatory effect. Phenol is an antioxidant because it contains a hydroxyl group that can absorb oxygen from free radicals, balancing oxygen production and reducing cell damage [28–30]. When the level of parasitemia is high, there is also a rise in the regulation of apoptosis-related genes because most apoptosis genes cease to be expressed with the degree of parasitemia [31].

During an infection, tumor necrosis factor-alpha (TNF-α) is secreted. Increasing ICAM-1 production enhances cytoadherence. TNF-α, when it binds to TNF receptor 1, sets off apoptosis through NF-kB activation [32, 33]. Neem leaf extract is believed to have the ability to block NF-B, which, in turn, lowers the production of proteins like ICAM-1 and stops infected red blood cells from adhering [34, 35]. Certain chemicals are found in the *A. indica* plant, particularly azadirachtin (a terpenoid), is thought to have antimalarial action. The growth of malaria parasites’ motile gametes can be inhibited by azadirachtin. There have been reports of the antiplasmodium activity of other substances, including steroids, alkaloids, flavonoids, and tannins [36]. Additionally, neem leaf extract has been shown to exhibit antimalarial activity, as evidenced by a reduction in parasitemia and brain hypoxia in cases of cerebral malaria [37].

## Conclusions

Malaria therapy in Indonesia has become resistant. Neem leaf extract can be utilized as an alternative to pharmaceuticals for the disease due to its diverse antimalarial properties, which include lowering parasitemia and preventing nerve cell death. The least amount of parasitemia (12 mg) and the least amount of brain neuron cell apoptosis (16 mg) were both most successfully reduced by neem leaf extract. These results offer a solid foundation for further research and the creation of anti-malarial medications. Clinical investigations and additional neem leaf toxicity testing are required.

## Authors’ Contributions

ZR and KCN: Conceptualized the study. AN, RR, and AM: Conducted the study, laboratory works, and data collection. NI and LFA: Sample collection. PA and MK: Sample collection, and performed data analysis. ZR, KCN, AN, RR, and AM: Supervised the study and wrote the original draft. ZR, PA, and MK: Reviewed and edited the manuscript. All Authors have read, reviewed, and approved the final manuscript.
